# Acute lower limb ischemia caused by vaccine-induced immune thrombotic thrombocytopenia: focus on perioperative considerations for 2 cases

**DOI:** 10.1186/s12959-022-00398-8

**Published:** 2022-07-04

**Authors:** Guillaume Roberge, Benoit Côté, Anthony Calabrino, Nathalie Gilbert, Nathalie Gagnon

**Affiliations:** 1 Centre d’Excellence Des Maladies Vasculaires, Centre Hospitalier Universitaire de Québec, Hôpital Saint-François d’Assise, Université Laval, Québec, Canada; 2 Department of General Internal Medicine, Centre Hospitalier Universitaire de Québec, Hôpital de L’Enfant-Jésus, Université Laval, Québec, Canada

**Keywords:** Vaccine-induced immune thrombotic thrombocytopenia, Heparin-induced thrombocytopenia, Arterial thrombosis, Limb ischemia, Perioperative care

## Abstract

**Background:**

ChAdOx1 nCoV-19 (AstraZeneca) and Ad26COV2.S (Johnson & Johnson/Janssen) adenoviral vector vaccines have been associated with vaccine-induced immune thrombotic thrombocytopenia (VITT). Arterial thrombosis and acute limb ischemia have been described in a minority of patients with VITT. These patients usually need a revascularization, but they potentially are at a higher risk of complications. Optimal perioperative care of patients undergoing vascular surgery in acute VITT is unknown and important considerations in such context need to be described.

**Cases presentations:**

We report 2 cases of VITT presenting with acute limb ischemia who needed vascular surgery and we describe the multidisciplinary team decisions for specific treatment surrounding the interventions. Both patients’ platelet counts initially increased after either intravenous immune globulin (IVIG) or therapeutic plasma exchange (TPE). None received platelet transfusion. They both received argatroban as an alternative to heparin for their surgery. Despite persistent positivity of anti-platelet factor 4 (PF4) antibodies and serotonin-release assay with added PF4 (PF4-SRA) in both patients, only one received a repeated dose of IVIG before the intervention. Per- and post-operative courses were both unremarkable.

**Conclusion:**

In spite of persistent anti-PF4 and PF4-SRA positivity in the setting of VITT, after platelet count improvement using either IVIG or TPE, vascular interventions using argatroban can show favorable courses. Use of repeated IVIG or TPE before such interventions still needs to be defined.

## Background

ChAdOx1 nCoV-19 (AstraZeneca) and Ad26COV2.S (Johnson & Johnson/Janssen) adenoviral vector vaccines have been associated with vaccine-induced immune thrombotic thrombocytopenia (VITT) [1–5]. This condition is similar to spontaneous heparin-induced thrombocytopenia (HIT), which is characterized by immune heparin-independent platelets activation involving anti-platelet factor 4 (PF4) antibodies (autoimmune HIT) [5, 6]. VITT is particularly associated with cerebral venous thrombosis and splanchnic vein thrombosis [1, 2, 5, 7]. However, a minority presents with lower limb ischemia caused by arterial thrombosis [1, 4, 5, 7, 8]. These patients usually require revascularization and management can become particularly challenging. Extrapolation from HIT data suggests they might have a higher bleeding risk due to thrombocytopenia, a potentially higher mortality and arterial thrombosis rate associated with platelets transfusion and higher thrombosis recurrence caused by the underlying immunological thrombotic process [9–11]. Optimal perioperative care of patients undergoing vascular surgery in acute VITT is unknown. We are describing 2 cases of lower limb ischemia diagnosed with this condition after receiving ChAdOx1 nCoV-19 vaccine. Both were tested negative for Covid-19. The first one has previously been described in a case series focusing on the role of therapeutic plasma exchange (TPE) in refractory VITT [12]. We are focusing here on perioperative management in VITT and outcomes related to vascular surgery.

### CASE 1

Patient 1 is a 48-year-old woman with past medical history of invasive ductal breast carcinoma in 2012, considered resolved but for which she is still taking tamoxifen. She consulted 16 days after vaccination, with 24 h of left lower limb pain. She had decreased mobility and sensitivity of the left foot. Her first three toes were cyanotic and distal pulses were absent. An intravenous heparin bolus was given before she was transferred to a tertiary vascular hospital.

**Fig. 1:**
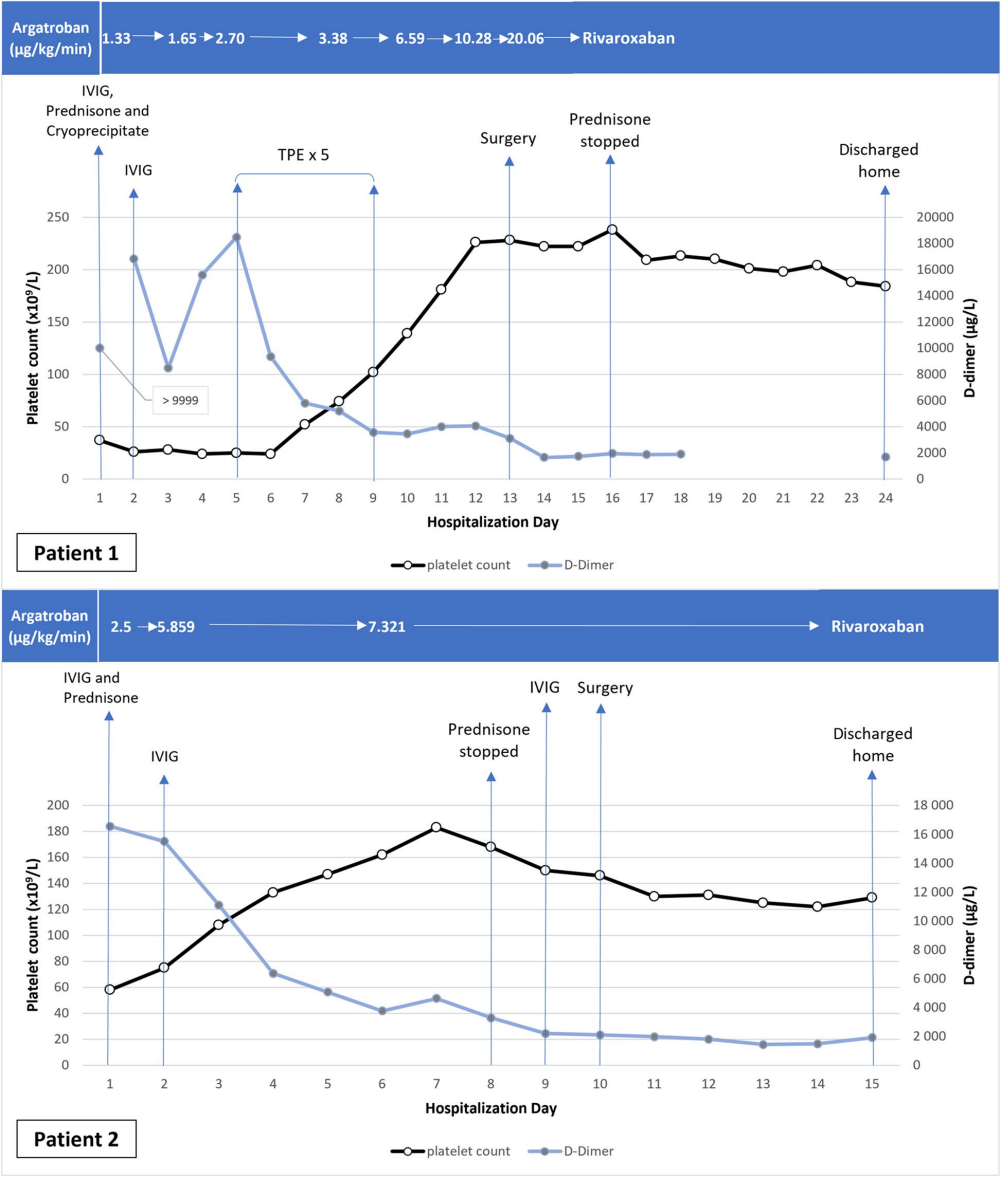
Platelet count and D-dimer evolution for 2 patients with acute VITT

On arrival, platelet count was 37 × 10^9^/L, INR 1.2, aPTT 24.5 s, fibrinogen 1.0 g/L, and D-dimer over 9999 µg/L (Fig. 1).

A CT scan demonstrated left subclavian artery thrombus, thoracic and abdominal aortic thrombus, total occlusion of the right internal iliac artery, and multiple thrombi in the left lower limb arteries without significant atherosclerosis. Troponin-I were 9713 ng/L (normal < 54 ng/L) and echocardiogram showed a 15–20% left ventricular ejection fraction with findings suggesting Takotsubo cardiomyopathy. VITT was suspected and argatroban was started at 0.5 mcg/kg/min, then progressively increased to achieve a target aPTT of 45-55 s (Fig. 1). Ten units of cryoprecipitate were also given as another fibrinogen level came back at 0.7 g/L. Polyspecific anti-PF4 immunoassay (Immucor) result was compatible with VITT (optical density (OD) 2.28). She later had a positive serotonin-release assay with added PF4 (PF4-SRA) performed at the McMaster Platelet Immunology Laboratory, which confirmed the diagnosis. She also received prednisone 1 mg/kg daily and intravenous immune globulin (IVIG) 1 g/kg (Panzyga®, 2 doses of 60 g, weight 68 kg) for 2 days. At this time, a multidisciplinary team of vascular medicine specialists, vascular surgeons, cardiologists and anesthesiologists decided not to proceed with urgent revascularization given cardiac instability and high perioperative risk.

**Fig. 2:**
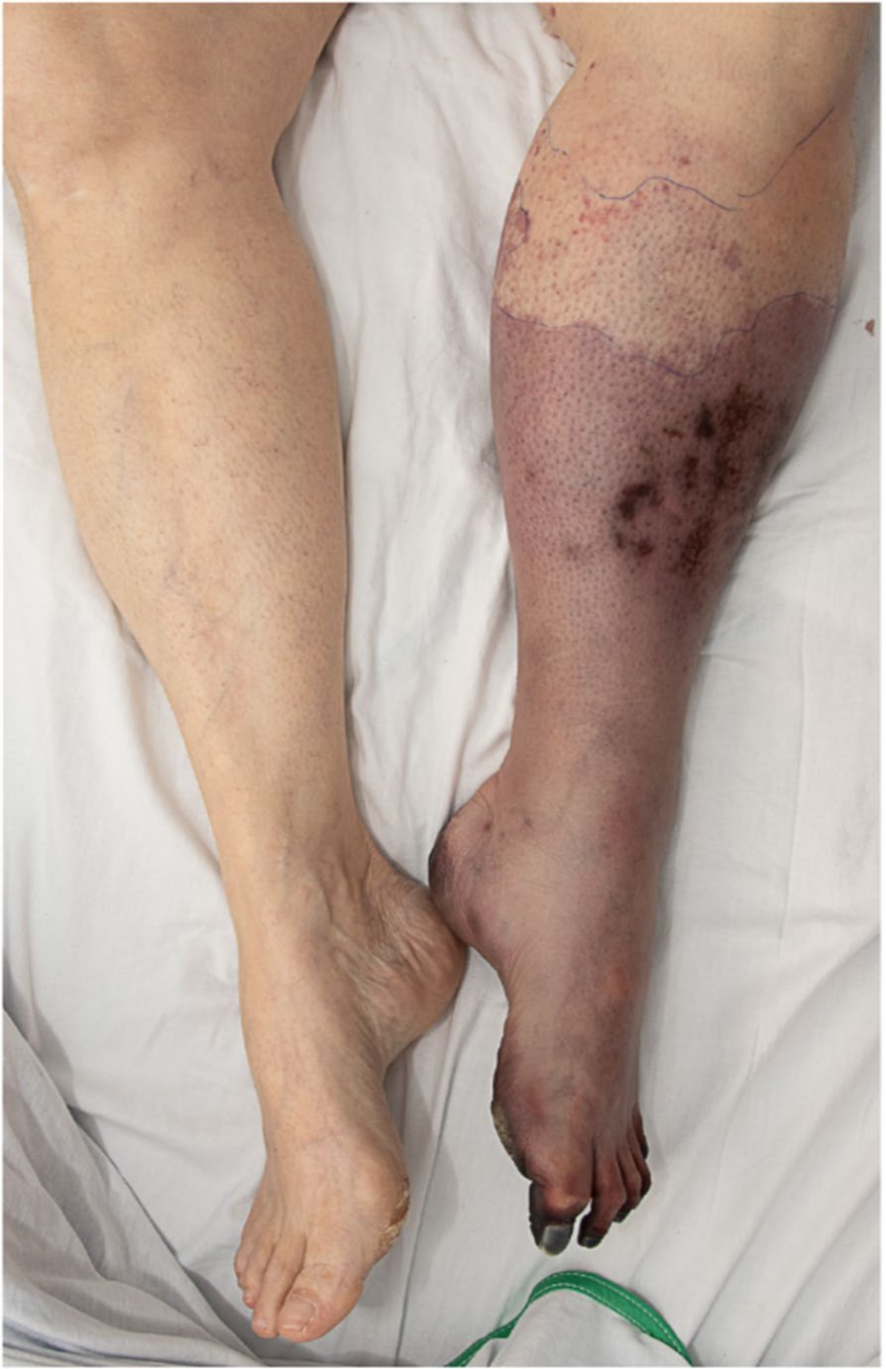
Left leg ischemia

On day 4, her left leg became more ischemic (Fig. 2). The platelet count was 24 × 10^9^/L and the D-dimer were increasing (15 596 µg/L). Reimaging showed new arterial thrombi in both legs and progression of the abdominal aortic thrombosis.

As this was considered a non-response to IVIG (no significant platelet count increase), TPE was started on day 5 for a total of five treatments. Argatroban perfusion was also empirically increased with aiming a higher aPTT target of 55-65 s. After the first TPE, platelet count increased and D-dimer went down and both continued to improve afterwards. On day 13, 4 days after the last TPE, platelet count was 228 × 10^9^/L and control PF4-ELISA OD was 1.43. At this time, she underwent an above-knee amputation with common femoral artery thrombectomy of her left lower limb without any complication. Argatroban dose during the surgery was 12.83 mcg/kg/min aiming a target aPTT of 65.1–80.0 s, and no bolus was needed. Per surgery, the vascular surgeon found a femoral vein thrombosis that had been previously absent. A few days later, she was switched to rivaroxaban 15 mg twice daily and discharged from the hospital.

### CASE 2

Patient 2 is a 56-year-old man with a past medical history of hypertension and hypercholesterolemia. He consulted 16 days after the first dose of ChAdOx1 nCoV-19 vaccine with a history of claudication of his left leg for the past 4 days and a constant new right calf pain. He also had a slight headache a few days before. Right leg edema and no distal pulses were noted on the left leg. Left popliteal pulse was preserved.

On arrival, platelet count was 58 × 10^9^/L, INR 1.1, aPTT 24 s, fibrinogen 1.3 g/L and D-Dimer 16 561 µg/L (Fig. 1). A right leg venous doppler ultrasound confirmed a great saphenous vein thrombosis with an extension to the femoral vein. CT angiogram showed an infrarenal aortic thrombus occluding 50% of the lumen and a left popliteal artery thrombosis without significant atherosclerosis. A CT pulmonary angiogram was done and revealed multiple segmental pulmonary embolisms. Brain imaging showed a cerebral vein thrombosis of the left sigmoid sinus with extension to the jugular vein without hemorrhage.

VITT was rapidly suspected and Polyspecific anti-PF4 immunoassay (Immucor) result came back positive (OD 2.13). The PF4-SRA, also performed at the McMaster Platelet Immunology Laboratory, was positive.

Patient was initiated on an argatroban dose of 2.0 mcg/kg/min adjusted to achieve an aPTT of 45-65 s (Fig. 1). He also received prednisone 1 mg/kg daily and IVIG 1 g/kg for 2 days (Panzyga®, 1 dose of 60 g and then 70 g, weight 65 kg). He remained stable after treatment initiation and the platelet count rapidly increased to 162 × 10^9^/L on day 6. On day 10, he had a left popliteal artery thromboembolectomy and patch angioplasty without any complication. The surgery was done aiming an aPTT of 45-65 s and using an argatroban dose of 7.321 mcg/kg/min. One bolus of 100 mcg/kg (6.5 mg) was needed. Since initial IVIG treatment had already been given several days before and because the PF4-ELISA result was still positive (OD 1.736), he received one additional dose of IVIG 1 g/kg (65 g) 12 h preoperatively. This was done even if the platelet count had remained stable (146 × 10^9^/L) and D-Dimer were going down. Four days after surgery, argatroban was switched to rivaroxaban 15 mg twice daily. Six days after the additional IVIG treatment, anti-PF4 was still positive (OD 1.855) but PF4-SRA was not tested. The patient was then discharged home.

## Discussion

Perioperative management of VITT cases presenting with acute limb ischemia is highly extrapolated from HIT and autoimmune HIT (aHIT) literature considering similar pathophysiology. Arterial thrombosis and acute limb ischemia have been well described in patients with usual HIT, but less with aHIT [13].

The first perioperative issue is the thrombocytopenia. Because of potential arterial thrombosis and mortality risk increase following routine platelet transfusions in HIT, they are usually avoided in VITT [10, 14, 15]. However, transfusions need to be considered for severe thrombocytopenia before urgent surgery when bleeding is expected [14, 15]. The usual risk–benefit balance in this context is compromised and optimal platelet count transfusion threshold before specific high-risk interventions in VITT is unknown.

Vascular interventions in acute HIT are underreported and the best perioperative anticoagulation regimen is also unknown. High intraoperative and early postoperative thrombosis recurrence rates have been described in patients with unrecognized acute HIT undergoing vascular surgeries while being initially treated with heparin [16, 17]. A report mentions that 14 vascular surgeries in 13 patients with active HIT were done in two hospitals over a period of 30 years. While two cases out of 10 performed using heparin needed subsequent amputation, three cases out of four done with non-heparin anticoagulation developed this unfavorable outcome. However, the difference was not statistically significant [18]. Another report mentions a case of aHIT developing after an abdominal aortic aneurysm repair for which a right iliac artery thromboembolectomy was done using argatroban with favorable outcomes [19]. However, the optimal argatroban dose and aPTT target in such surgery are unknown.

Considering limited data, IVIG and TPE seems to adequately raise platelet count in HIT. [20]. According to a national HIT database from which 77 and 52 patients were treated with TPE and IVIG respectively, outcomes after one or the other were similar [21]. IVIG have been more described in aHIT and show variable but favorable response, usually defined by an increase of platelet count by ≥ 50 × 10^9^ /L within 5 days [22]. IVIG efficacy in VITT is also favorable but with fluctuating anti-PF4 and serotonin release response [8]. TPE in refractory VITT is also recommended and has also shown beneficial results [7, 12]. Both modalities are recommended lines of treatment in VITT.

Preoperative IVIG have been described in active HIT for a left femoral-popliteal artery bypass using intraoperative heparin. The serotonin-release assay became negative for 2 days after IVIG and came back to its maximal activity after 7 days [23]. TPE has been more described in the setting of cardiac surgeries for patients with active HIT necessitating heparin. A case described by Warkentin et al. underwent 4 preoperative TPE treatments before cardiac surgery while exposed to heparin and resulted in no subsequent increase in anti-PF4 antibodies level for at least 7 days [24]. Current guidelines recommend preoperative or intraoperative TPE for patients with acute HIT who need cardiovascular surgery and heparin exposure [14]. No recommendation is established regarding perioperative IVIG. TPE and IVIG potential preoperative role before vascular interventions in acute aHIT are not defined. In VITT, the heparin-independent anti-PF4 activity also brings uncertainty regarding bleeding and thrombosis recurrence after such high-risk procedures.

In a prospective cohort of 220 VITT cases, aortic thrombosis and ischemic limb were reported in 12% [7]. A VITT case presenting with acute left leg ischemia has showed successful initial left lower limb thrombectomy done on heparin [25]. However, the VITT diagnosis was not initially suspected, and the patient was treated with heparin and also received platelet transfusions. Such as routine platelet transfusions, heparin is not recommended in VITT considering extrapolation from HIT data [10, 14, 26]. The patient then received the IVIG and fondaparinux when VITT was diagnosed, but then suffered a thrombosis recurrence and amputation. Two other cases underwent left leg thrombectomy before receiving IVIG. One was presumably done with argatroban, while the other with heparin, and is awaiting amputation because of residual necrotic ischemia [8].

Our first case, initially described in another publication, was deemed too unstable to undergo a revascularization because of her cardiac condition and a decision was made not to proceed with surgery [12]. She was unresponsive to recommended IVIG therapy and had five subsequent TPE. With expected progression of the leg ischemia but improvement of her global condition after TPE, an above-knee amputation and common femoral artery thrombectomy was done 4 days after the last TPE. Argatroban was continued during the procedure and a higher aPTT target was empirically chosen to avoid thrombosis progression as she was deemed at high risk considering the severity of the clinical presentation. Anti-PF4 was still positive (OD 1.43) the day before the intervention. Despite this anti-PF4 persistence, no supplemental preoperative IVIG or TPE was used considering that the last TPE treatment was done four days before and the platelet count was improving. PF4-SRA was still positive retrospectively. Postoperative evolution was favorable.

Our second case also had his left leg thromboembolectomy initially postponed, but it was because the limb was not threatened. He received IVIG with significant platelet count improvement. Because the anti-PF4 OD was still high and the last IVIG received was 7 days before, and considering potential increase in SRA activity in HIT after such a delay [23], we felt more comfortable to give another IVIG dose 12 h preoperatively. The intervention was done using argatroban aiming usual aPTT target, without significant bleeding. The clinical course was favorable despite PF4-SRA still being positive retrospectively.

In both cases, high argatroban doses were progressively required to maintain the aPTT in therapeutic range. Higher infusion rates compared to usual recognized effective ones in HIT (1.6–2.1 μg/kg/min to achieve target aPTTs of 1.5–3 times the baseline value) were necessary and the maximum recommended dose of 10 μg/kg/min was exceeded for case 1 [27]. It is unknown if an underlying coagulopathy specifically associated with VITT could explain this phenomenon. This may also reflect the possible moderate linear correlation between high-dose argatroban and aPTT time and concerns of potential high argatroban serum concentration not reflected by the aPTT. Despite the use of such high doses, no significant bleeding occurred, aPTTs were in therapeutic range and higher target was not used (except for the time of surgery for case 1). In spite of not being a recognized line of treatment in HIT, corticosteroids were also used as recommended in VITT, based on potential antibodies production suppression such as seen in immune thrombocytopenia [14, 15, 28]. For both patients, need for preoperative optimization of disease activity and best expected timing for surgery were extrapolated from limited HIT and aHIT data. Considering more available prospective data with rivaroxaban in HIT compared to others direct oral anticoagulants, it was chosen for anticoagulation after the surgeries in both cases [29]. Warfarin was also avoided to minimize potential anticoagulation fluctuations. Per- and post-operative courses were both unremarkable.

## Conclusion

Our two cases highlight important perioperative aspects of VITT presenting with acute lower limb ischemia. They suggest argatroban as a potential safe alternative to heparin for vascular surgery in such patients. They also suggest possible safety of vascular interventions after initial stabilization and platelet count improvement using either IVIG or TPE, despite persistent positivity of anti-PF4 and PF4-SRA. Use of repeated IVIG or TPE before such interventions still needs to be defined.

## Data Availability

Data sharing is not applicable to this article as no datasets were generated or analysed during the current study.

## Abbreviations

VITT: Vaccine-induced immune thrombotic thrombocytopenia
HIT: Heparin-induced thrombocytopenia
PF4: Platelet factor 4
TPE: Therapeutic plasma exchange
OD: Optical density
PF4-SRA: Serotonin-release assay with added PF4
IVIG: Intravenous immune globulin
aHIT: Autoimmune HIT
